# GIT1 protects traumatically injured spinal cord by prompting microvascular endothelial cells to clear myelin debris

**DOI:** 10.18632/aging.202560

**Published:** 2021-02-17

**Authors:** Bowen Wan, Cong Li, Ming Wang, Fanqi Kong, Qirui Ding, Chenliang Zhang, Hao Liu, Dingfei Qian, Wenlin Deng, Jian Chen, Pengyu Tang, Qian Wang, Shujie Zhao, Zheng Zhou, Tao Xu, Yifan Huang, Jun Gu, Jin Fan, Guoyong Yin

**Affiliations:** 1 Department of Orthopedics, The First Affiliated Hospital of Nanjing Medical University, Nanjing 210029, China; 2 Department of Plastic and Burn Surgery, The First Affiliated Hospital of Nanjing Medical University, Nanjing 210029, China; 3 Department of Orthopedics, Changzheng Hospital, The Second Military Medical University, Shanghai 200003, China; 4 Department of Orthopedics, The Affiliated Shuyang Hospital of Xuzhou Medical University, Suqian 223600, China; 5 Department of Orthopedics, The Affiliated Suqian First People's Hospital of Nanjing Medical University, Suqian 223800, China; 6 Department of Orthopedics, Xishan People's Hospital, Wuxi 214000, China

**Keywords:** GIT1, myelin debris, autophagy, angiogenesis, spinal cord injury

## Abstract

The clearance of myelin debris is a critical step in the functional recovery following spinal cord injury (SCI). As phagocytes do, microvascular endothelial cells (MECs) participate in myelin debris clearance at the injury site within one week. Our group has verified that G protein-coupled receptor kinase 2 interacting protein-1 (GIT1) is essential in autophagy and angiogenesis, both of which are tightly related to the uptake and degradation of myelin debris by MECs. Here, we analyzed the performance and mechanism of GIT1 in myelin debris clearance after SCI. The SCI contusion model was established and *in vitro* MECs were treated with myelin debris. Better recovery from traumatic SCI was observed in the GIT1 WT mice than in the GIT1 KO mice. More importantly, we found that GIT1 prompted MECs to clear myelin debris and further enhanced MECs angiogenesis *in vivo* and *in vitro*. Mechanistically, GIT1-mediated autophagy contributed to the clearance of myelin debris by MECs. In this study, we demonstrated that GIT1 may prompt MECs to clear myelin debris via autophagy and further stimulate MECs angiogenesis via upregulating VEGF. Our results indicate that GITI may serve as a promising target for accelerating myelin debris clearance and improving SCI recovery.

## INTRODUCTION

As contusive SCI occurs, myelin sheaths are mechanically compressed and demyelination becomes prominent. In addition, myelin loss, axonal damage, and microvasculature disruption, can counter tissue regeneration and functional recovery [1–3]. As myelin sheaths fall apart after SCI, myelin debris aggregates in the injury site and releases deleterious molecules to disrupt axon regeneration and remyelination [4–7]. Therefore, myelin debris should be cleared away from the injury site prior to SCI repair.

Though myelin debris can generally be swept away by “professional” phagocytes like bone marrow-derived macrophages (BMDMs) and resident microglia [8–10], it takes at least one week for BMDMs to converge into the SCI epicenter, a site deprived of microglia [8, 9, 11]. Microvessels and MECs assemble in the injury core one day after SCI, till to a normal level within one week [12, 13]. Previous studies proved that MECs could support myelin debris clearance shortly after SCI [14].

GIT1 cooperates with G-protein-coupled receptor kinase 2 (GRK2) in the endocytosis of adrenergic receptors [15, 16]. Once tightened with signaling molecules, GIT1 takes on various physiological profiles [15–17]. We have found that GIT1 is engaged in the development of pulmonary vessels [18] and CD31^hi^Emcn^hi^ vessels in fracture healing [19]. In terms of bone homeostasis, GIT1 contributes to autophagy in starved osteoclasts [20]. Given these, we hypothesize that GIT1 could act in autophagy to prompt MECs to clear myelin debris after SCI, which has been proven by the current study.

## RESULTS

### GIT1 expression is upregulated in the spinal cord after SCI

**Figure 1:**
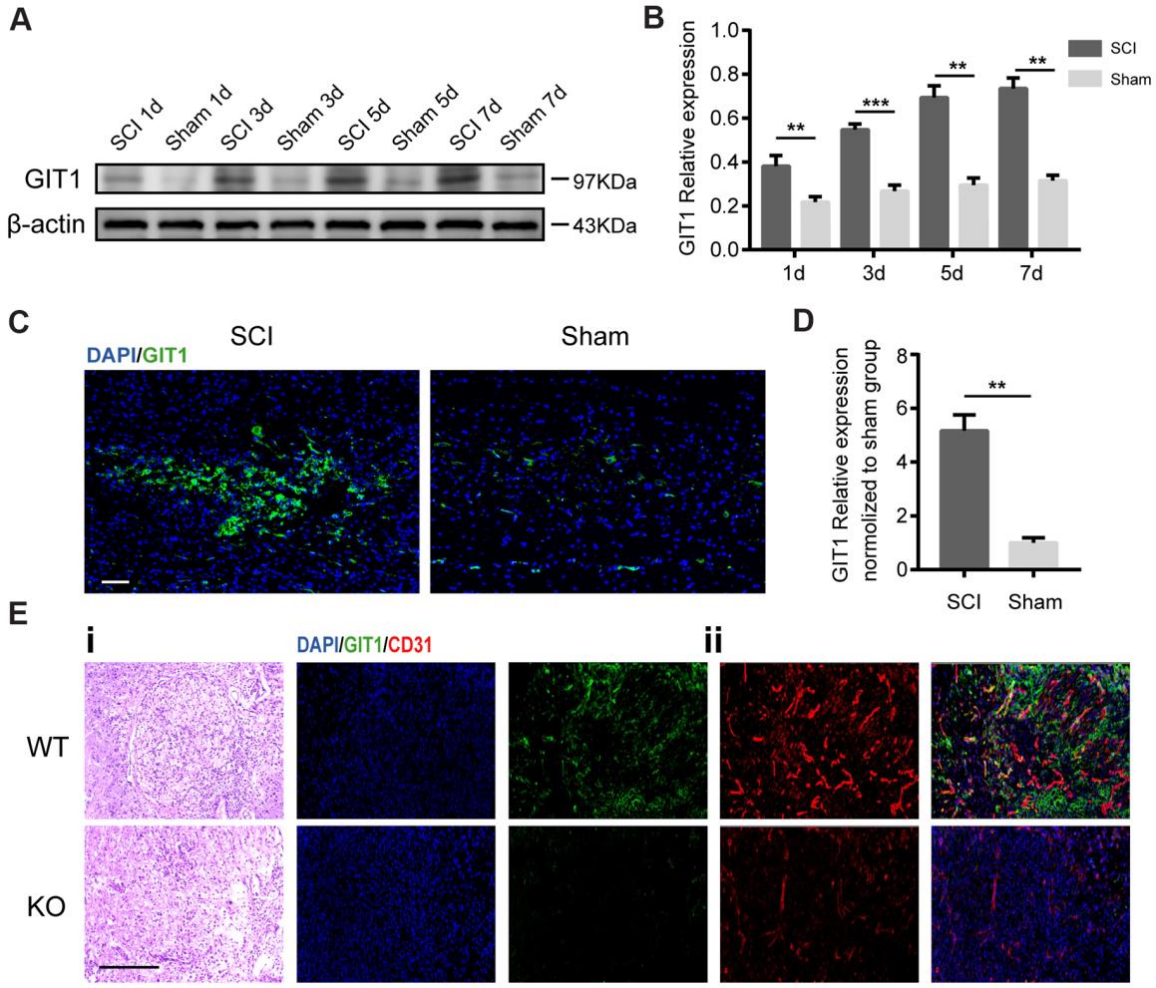
**GIT1 is highly expressed *in vivo* after SCI.** (**A**, **B**) Western blots and semiquantification of GIT1 at each time point after SCI or sham surgery. (**C**, **D**) Immunofluorescence images for GIT1 (green) and quantification at day 7 after SCI or sham surgery. Nuclei were counterstained using DAPI (blue). Scale bar, 100 μm. (**E**) Spinal cord at 7 days post-SCI: (**i**) representative H&E images in the lesion site from the GIT1 WT and KO groups. Bar, 100 μm. (**ii**) IF staining of GIT1 (green) and CD31 (red) in the lesion epicenter from the GIT1 WT and KO groups. Nuclei were stained using DAPI (blue). Scale bar, 100 μm. N = 5 animals per group. ^***^p < 0.01, ^***^p < 0.001.

To investigate the role of GIT1 in the pathophysiology of SCI, western blot analysis was employed to quantify GIT1 expression at 1, 3, 5, 7 days post-SCI. Immunofluorescence staining was performed at day 7 after SCI. The protein level of GIT1 in SCI group increased significantly and peaked at day 7 (Figure 1A–1D), mainly in microvascular endothelial cells (Figure 1E).

### GIT1 protects against SCI-induced spinal cord damage

**Figure 2:**
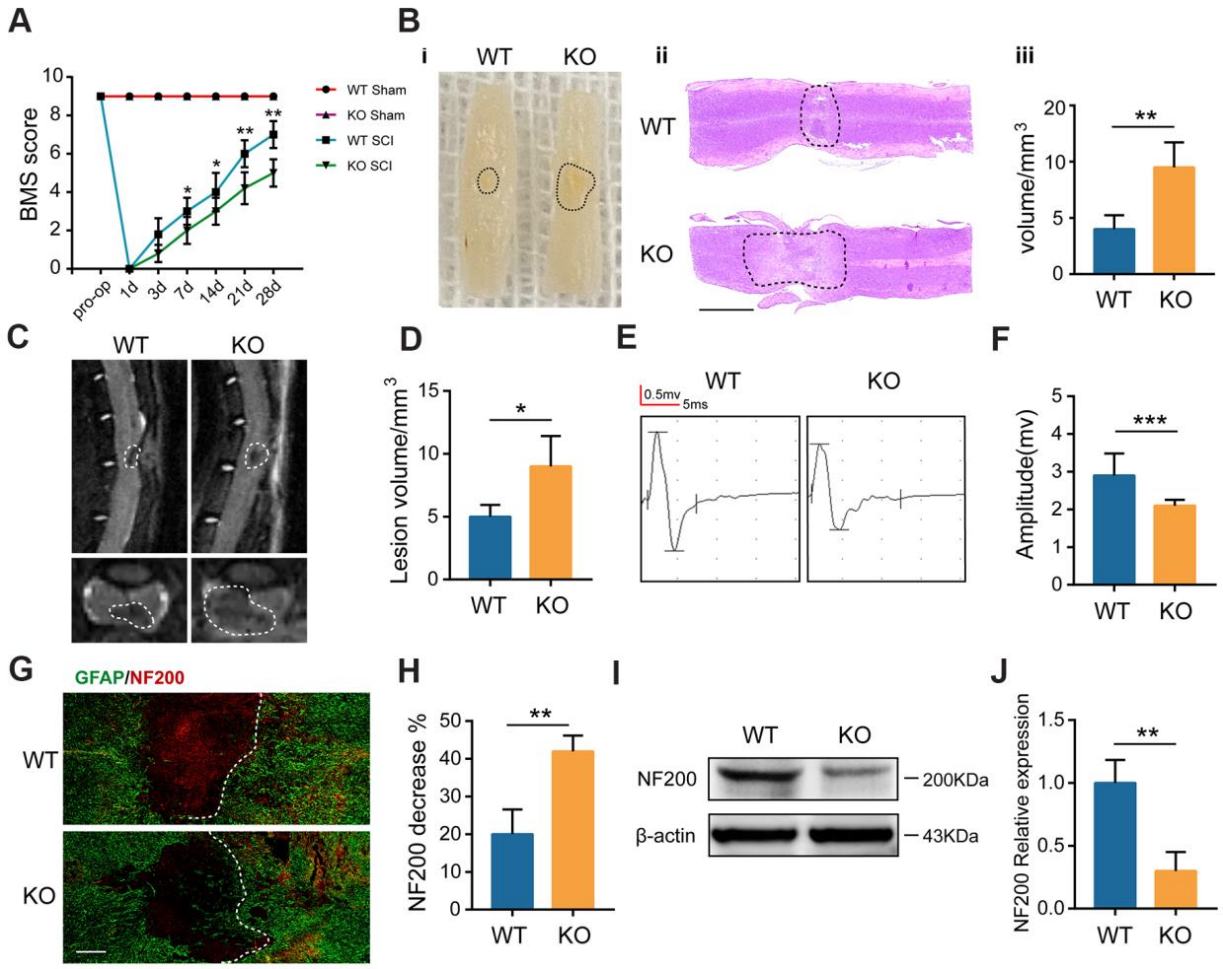
**GIT1 deficiency exacerbates SCI-induced spinal cord damage.** (**A**) Basso Mouse Scale results over 28 days. (**B**) Spinal cord at 28 days post-SCI: (**i**) gross morphology, (**ii**) representative H&E stained sections. Scale bar = 2000 μm, and (**iii**) the lesion volumes. (**C**, **D**) Sagittal and axial spinal cord T2 weighted images at day 28 after SCI. (**E**, **F**) MEP analysis. (**G**, **H**) IF staining of GFAP (green) and NF200 (red) at the injury sites at 7 days after SCI. Nuclei were stained using DAPI (blue); the dashed lines represent the boundary of the injury area. Scale bar = 100 μm. (**I**, **J**) Representative western blots of NF200 and the semiquantification of relative expression levels of NF200. N = 6 animals in each group. ^*^p < 0.05, ^**^p < 0.01, ^***^p < 0.001.

The Basso Mouse Scale (BMS) was first employed to assess the functional recovery in both GIT1 WT and GIT1 KO mice after SCI. As revealed in Figure 2A, no obvious between-group difference showed up in the BMS scores before SCI. In contrast, score of WT mice began to exceed that of KO mice at day 7, till day 28 after SCI. The gross morphology of traumatic lesion site was clear at day 28 after SCI. However, compared with the GIT1 KO mice, the lesion area was notably smaller in the GIT1 WT mice. The H&E staining results further indicated that the GIT1 KO group displayed more severe destruction of central gray matter and peripheral white matter, while the GIT1 WT group had a decreased cavity of necrotic tissue around the injury site (Figure 2B). We further examined the pathological change of spinal cord at 28 days after SCI. The damaged volume was illustrated in Figure 2C and the MRI results verified that GIT1 deficiency remarkably increased the necrotic volume compared with the GIT1 WT group (Figure 2D). According to electrophysiological analyses (Figure 2E, 2F), MEP amplitudes in the WT group rose to a higher level than that in the KO group at day 28 post-SCI, indicating the better recovery of the hindlimbs in WT mice. Finally, we examined the abundance of neurons and axons with 200 kDa subunit of neurofilament (NF200). During the scar formation post-SCI, the astrocyte hypertrophies and glial fibrillary acidic protein (GFAP) increases. The reactive astrocytes were visualized using GFAP antibody. As shown in Figure 2G, 2H, compared with that in the neighboring areas, the pixel intensity of NF200 decreased more significantly in the lesion site in the KO group than in the WT group at 7 days after SCI. As indicated by western blot images in Figure 2I, 2J, GIT1 remarkedly protected neurons and axons at day 7 after SCI. The western blot results were in high accordance with the immunofluorescence results, both indicating that GIT1 protected neurons as well as axons, and ultimately promoted functional behavioral recovery following SCI.

### GIT1 participates in myelin debris clearance in the injured spinal cord

**Figure 3:**
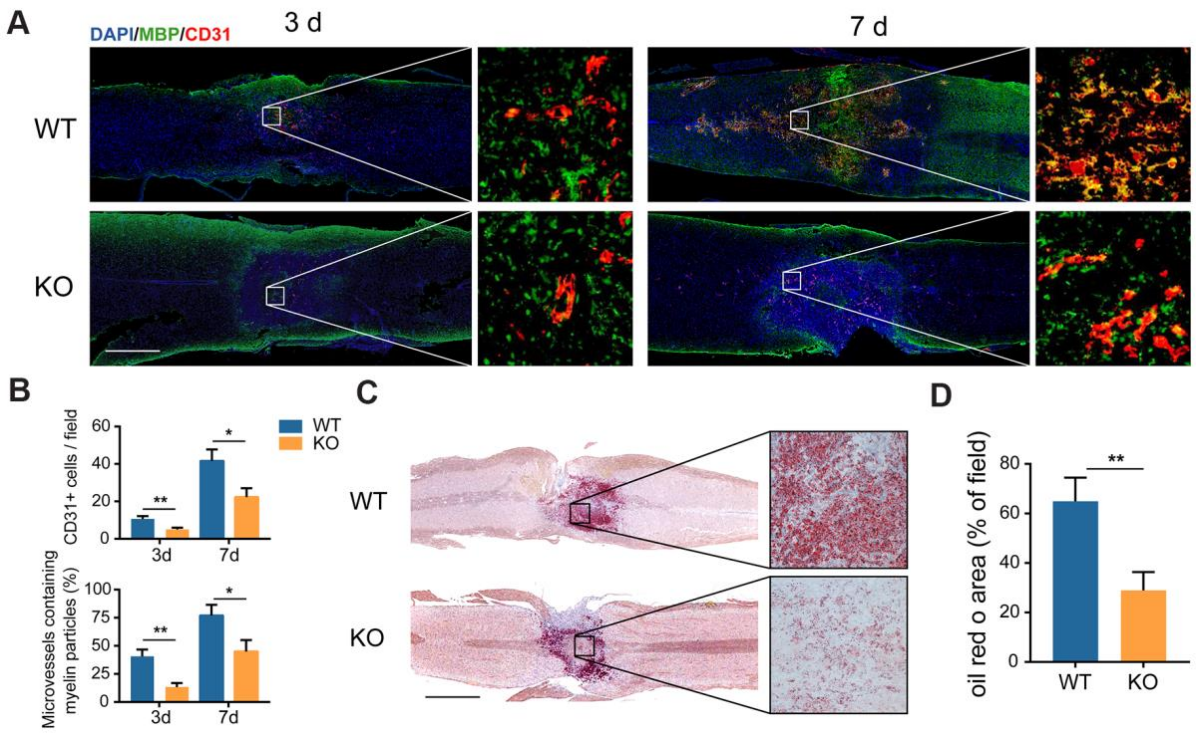
**The clearance of myelin debris is reduced in the GIT1 KO mice after SCI.** (**A**) IF staining of myelin debris marker MBP (green) and MECs marker CD31 (red) at the injury core of the GIT1 WT and KO mice at 3 and 7 days after SCI. Nuclei were stained using DAPI (blue). Bar = 500 μm. (**B**) Quantification of CD31-postive MECs and myelin-containing MECs in the injury sites at day 3 and 7 post-SCI. (**C**) Oil Red O images at day 7 post-SCI. Bar = 2000 μm. (**D**) Quantification of the ORO stained positive areas in the GIT1 WT and KO groups. N = 6 animals per group. ^*^p < 0.05, ^**^p < 0.01.

It has been revealed that MECs play the role of phagocytes for myelin debris clearance at the injury site after SCI [14]. In the present study, the GIT1 KO mice were generated to explore whether GIT1 is involved in this process. Microvessels in the lesion epicenter disappeared quickly following SCI, whereas MECs rose at the injury core one day after SCI, and fell to the normal level within one week (Supplementary Figure 1A, 1B). We first tested whether new microvessels and MECs could swallow myelin debris. As revealed in Figure 3A, 3B, MBP started to bind to newly formed microvessels at the lesion core at day 3, which became more pronounced at day 7 after SCI. More importantly, microvessels in the lesion site from the GIT1 WT mice contained much more detectable myelin debris (MBP positive) compared with the GIT1 KO mice at 3 and 7 days post-SCI, suggesting that phagocytic activity weakened in the GIT1 KO mice. Once engulfed, myelin debris is degraded into neutral lipids in lysosomes [8]. Therefore, these neutral lipids could be detected with oil red O (ORO) staining [21]. We also found that the ORO stained positive areas in the GIT1 WT group were significantly larger than those in the GIT1 KO group at day 7 post-SCI, indicating that less myelin debris was degraded in the mice lacking GIT1 (Figure 3C, 3D). Based on these results, we believed that GIT1 deficiency may impair the ability of MECs to engulf and degrade myelin debris *in vivo*.

### GIT1 promotes MECs growth in the injured spinal cord

**Figure 4:**
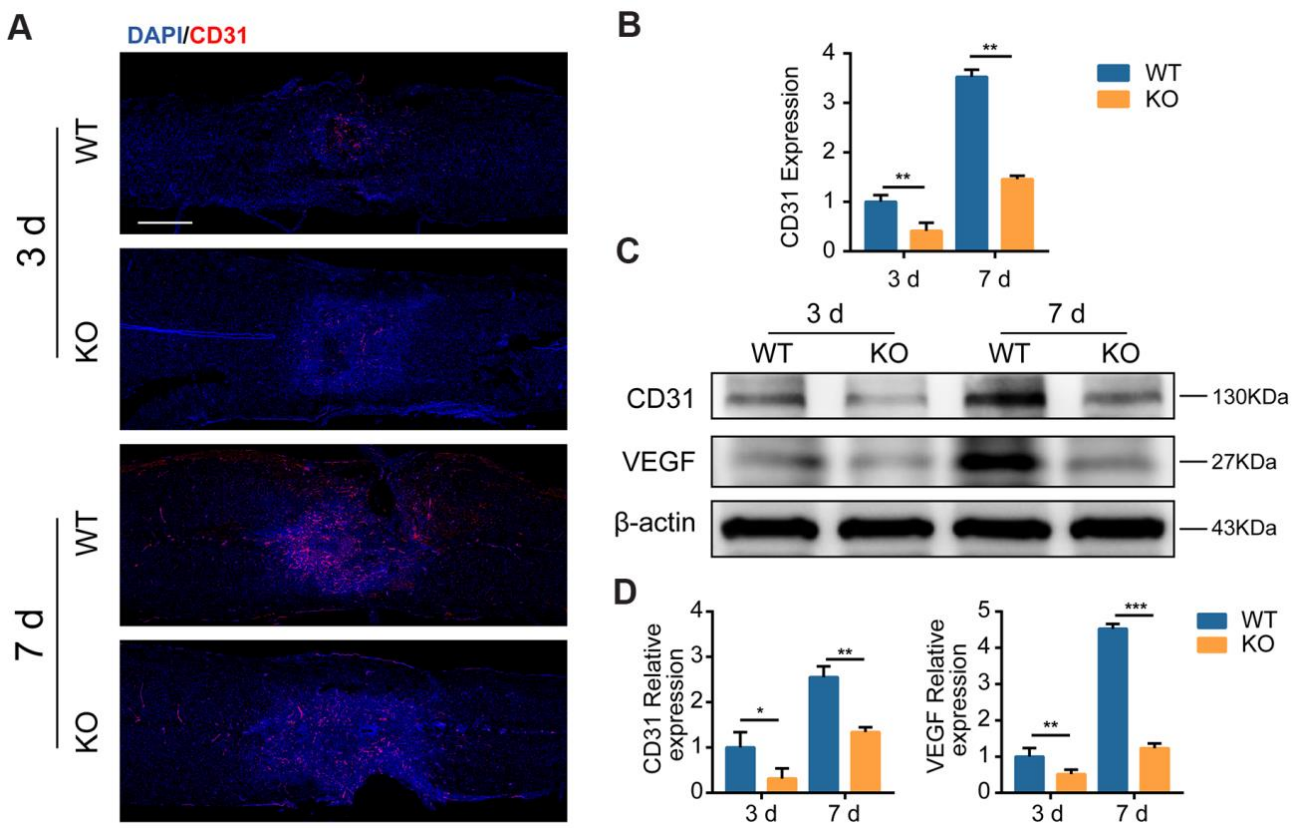
**GIT1 promotes MECs angiogenesis *in vivo*.** (**A**, **B**) Representative IF and quantification of microvessels and MECs at the injury core in the GIT1 WT and KO groups at day 3 and 7 after SCI. Nuclei were counterstained with DAPI (blue). Bar = 500 μm. (**C**, **D**) Representative western blots of CD31 and VEGF and the semiquantification of relative expression levels of CD31 and VEGF. N = 5 animals in each group. ^*^p < 0.05, ^**^p < 0.01, ^***^p < 0.001.

Next, we sought to identify whether GIT1-related myelin debris engulfment and degradation could stimulate MECs growth. CD31 positive microvessels decreased in the GIT1 KO group at the lesion epicenter compared with the GIT1 WT group at day 3 and 7 post-SCI (Figure 4A, 4B). Western blot images showed that at day 3 and 7 after SCI, the expression of CD31 and VEGF were markedly decreased in the GIT1 KO group compared with the GIT1 WT group (Figure 4C, 4D). The above results suggested that GIT1 benefitted myelin debris clearance by MECs, and may further promote MECs angiogenesis via increasing VEGF expression in the injured spinal cord.

### GIT1 enhances myelin debris clearance *in vitro*

**Figure 5:**
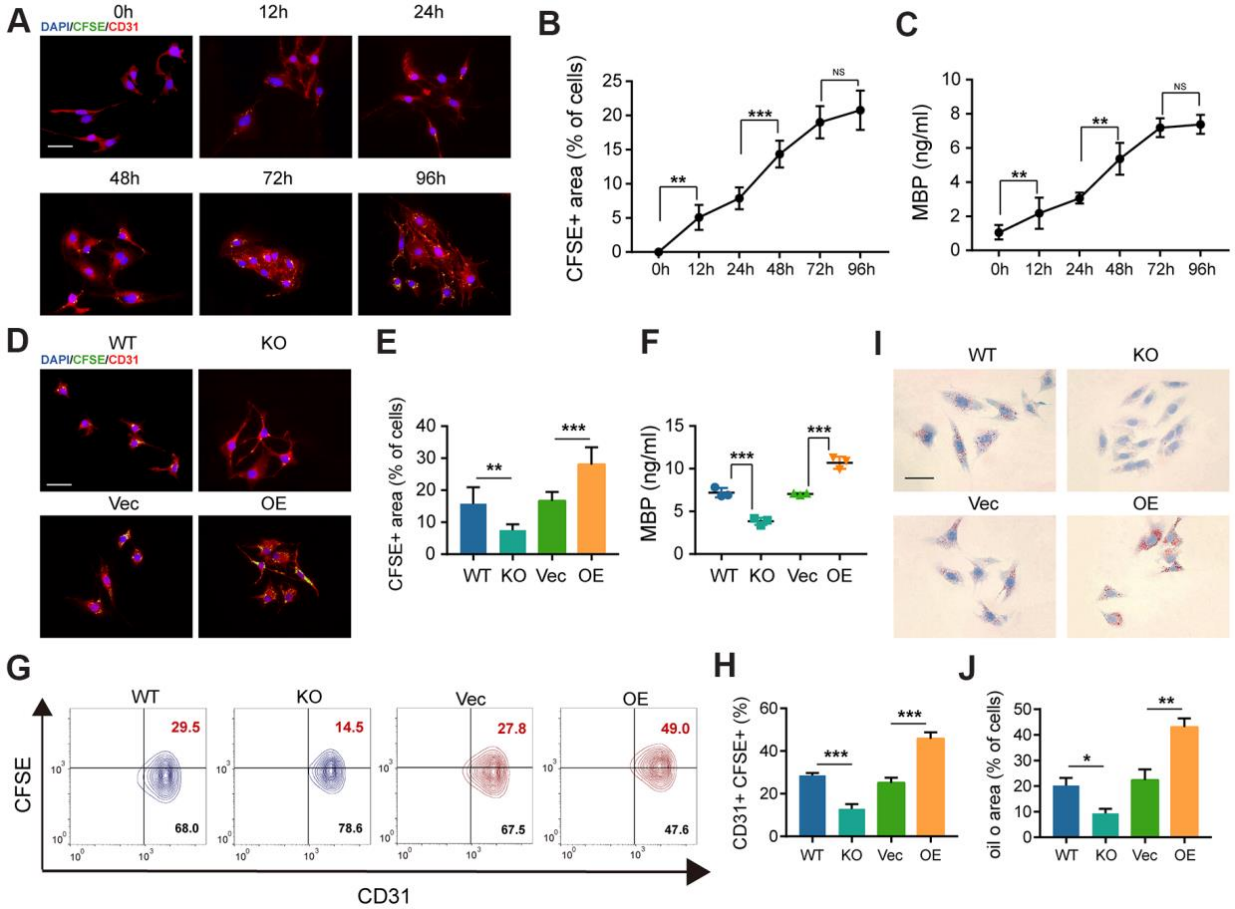
**The deficiency of GIT1 in BMECs fails to engulf and degrade myelin debris efficiently *in vitro*.** (**A**) Representative IF images showing CFSE-labeled myelin debris (green) engulfed by BMECs (red) at indicated time points. Nuclei were stained using DAPI (blue). Scale bar = 20 μm. (**B**) Phagocytosis of myelin debris (the percentage of the CFSE positive area). (**C**) ELISA results of intracellular MBP after being cultured with or without myelin debris at indicated time points. (**D**, **E**) IF staining and quantification of phagocytosis of CFSE-labeled myelin debris by BMECs at 72 h. Scale bar = 20 μm. (**F**) ELISA detection of intracellular MBP in different BMECs after being cultured with myelin debris for 72 h. (**G**, **H**) FACS detection of myelin-laden BMECs at 72 h. The CD31+ and CFSE+ quadrant represents myelin-laden BMECs. (**I**) Oil Red O staining of BMECs in the different groups after being cultured with myelin debris for 72 h. Scale bar = 20 μm. (**J**) Quantification of the ORO stained positive areas of BMECs from the different groups after being stimulated with myelin debris for 72 h. N = 6 in each group. NS represents no significance, ^*^p < 0.05, ^**^p < 0.01, ^***^p < 0.001.

To investigate whether GIT1 influences MECs in clearing myelin debris *in vitro*, we used the primary mouse brain microvascular endothelial cells (BMECs) from GIT1 WT and KO mice and overexpressed GIT1 in BMECs. The efficiencies of GIT1 knockout and overexpression were verified by western blot assays (Supplementary Figure 2A, 2B). First, the pieces of myelin debris were tracked after having been labeled with carboxyfluorescein succinimidyl ester (CFSE) [22]. Then, the CFSE-labeled myelin debris was incubated with the primary mouse BMECs, and the internalized pieces were displayed as scattered puncta within the cytoplasm using the fluorescence microscope. As shown in Figure 5A, the primary mouse BMECs ingested myelin debris gradually, till perinuclear distribution became predominant. After incubation with myelin debris, the percentage of CFSE-positive area and the concentration of intracellular MBP were quantified by immunofluorescence assessment and ELISA analysis (Figure 5B, 5C). The myelin debris engulfment by BMECs became inefficient during 12-48 h, efficient during 48-72 h and saturated during 72-96 h (Figure 5A–5C). More importantly, the primary mouse BMECs from the GIT1 KO mice brought forth a small population positive to CFSE, compared to those from the GIT1 WT mice, and more phagocytosed CFSE-labeled myelin debris in the GIT1-OE group than in the control group, after 72 h’s treatment with myelin debris (Figure 5D, 5E). The percentage of cells positive to both CD31 and CFSE, and the concentration of intracellular MBP showed the same changes (Figure 5F–5H). Oil Red O staining was used to evaluate myelin debris degradation, with BMECs from the GIT1 WT and GIT1-OE groups showing more ORO positive lipid droplets within the cytoplasm (Figure 5I, 5J), verifying that GIT1 deficiency had not degraded the myelin debris into neutral lipids. The above results demonstrated that GIT1 contributed to the clearance of myelin debris by BMECs *in vitro*.

### GIT1 promotes BMECs proliferation and angiogenesis *in vitro*

**Figure 6:**
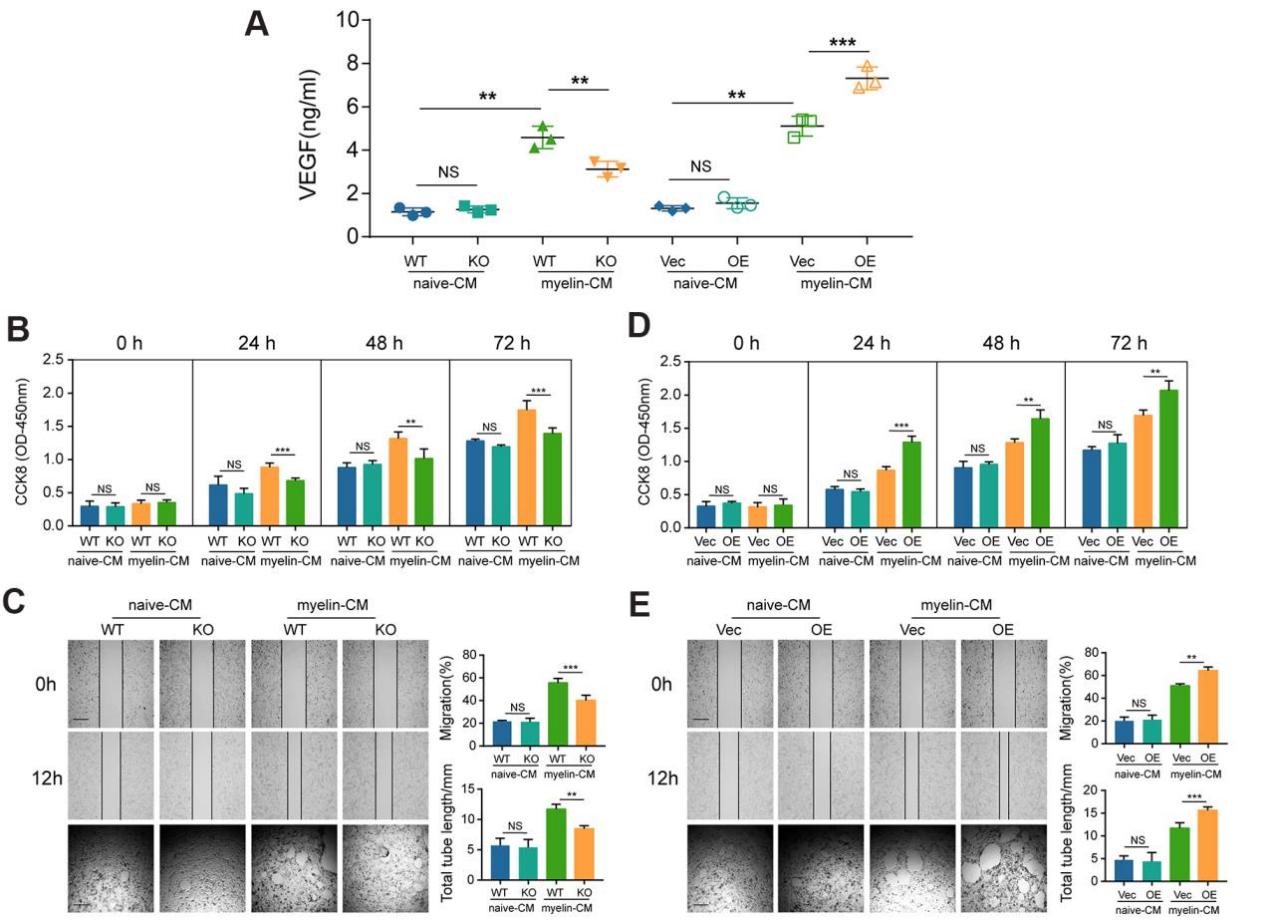
**GIT1 deficiency inhibits BMECs proliferation and angiogenesis *in vitro*.** (**A**) ELISA detection of VEGF in the CM of BMECs from the different groups after being treated with or without myelin debris for 72 h. (**B**, **D**) Proliferation of BMECs cultured with CM from the different groups examined by CCK8 at 0, 1, 2, and 3 d. (**C**, **E**) Migration and tube formation of BMECs treated with CM from the different groups. The images of scratch wound and tube formation assays (left) and quantification of the migration ability and cumulative tube length (right). Scar bar, 100 μm. N = 6 in each group. NS represents no significance, ^*^p < 0.05, ^**^p < 0.01, ^***^p < 0.001.

VEGF plays a critical role in angiogenesis [23]. As already illustrated in Figure 4C and 4D, GIT1 increased the expression of VEGF in the injured cord. Next, we sought to identify whether GIT1 influences BMECs proliferation and angiogenesis *in vitro*. As demonstrated in Figure 6A, the concentration of VEGF in the CM of BMECs from the different groups were significantly upregulated after being treated with myelin debris for 72 h. Moreover, myelin-induced VEGF expression was partly abrogated after GIT1 knockout, indicating that the engulfment and degradation of myelin debris are indispensable for the secretion of VEGF by BMECs. CM from the GIT1 WT group, having been stimulated with myelin debris for 72 h, could increase BMECs proliferation, migration and tube formation, compared to CM from the GIT1 KO group (Figure 6B, 6C). Similar results were observed in BMECs cultured with CM that had been treated with myelin debris for 72 h in the GIT1-OE group, compared to CM from the control group (Figure 6D, 6E). These data were highly consistent with the results *in vivo*, collectively indicating that GIT1-related myelin debris engulfment and degradation may stimulate BMECs proliferation and angiogenesis. The proangiogenic potential may partially be attributed to the increased expression of VEGF.

### GTI1 prompts BMECs to clear myelin debris via autophagy pathway

**Figure 7:**
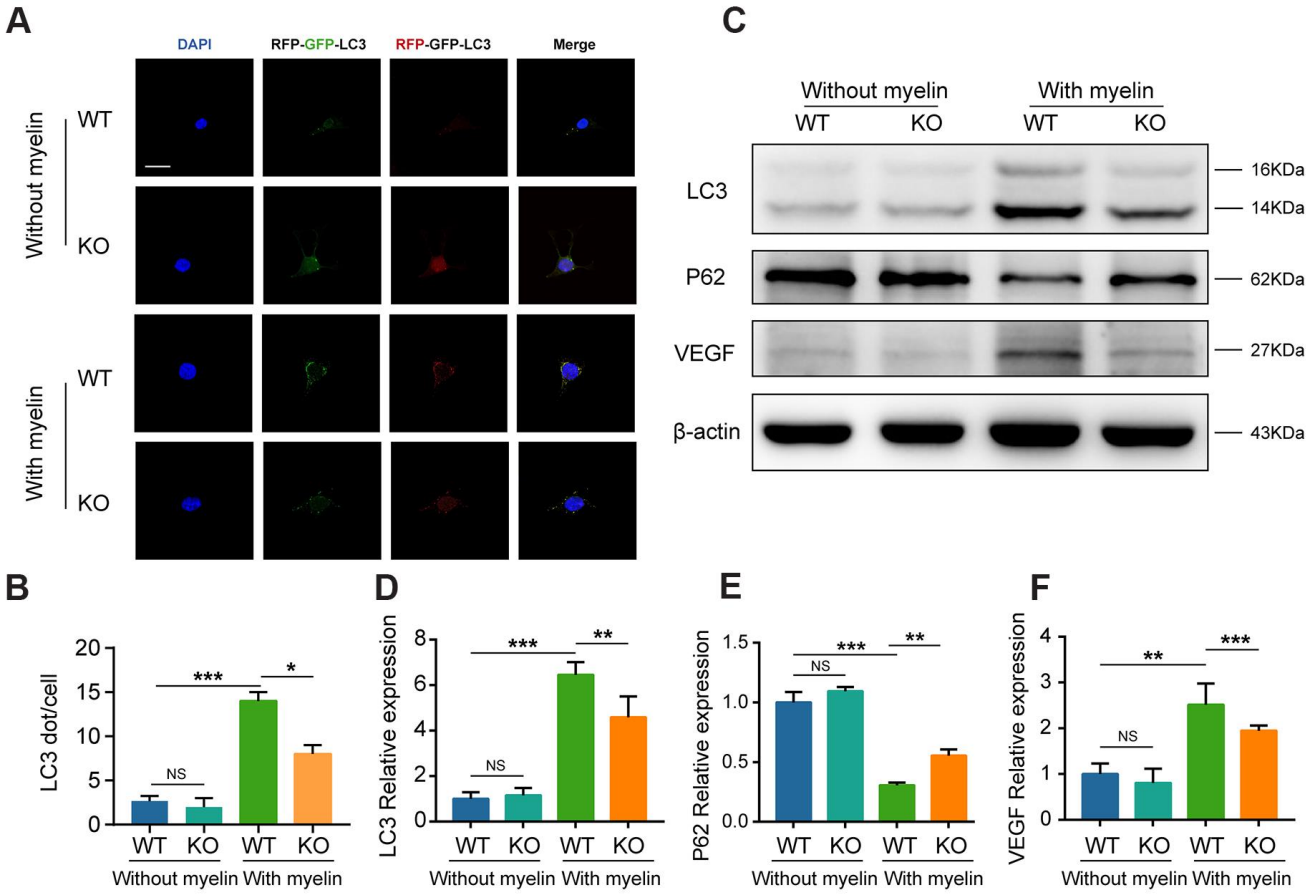
**GTI1 promotes MECs to clear myelin debris via autophagy pathway.** (**A**) BMECs from the GIT1 WT and KO mice were transfected with the mRFP-GFP-LC3 adenovirus. The cells nuclei were stained using DAPI (blue). Bar, 10 μm. (**B**) Numbers of yellow and red puncta. (**C**–**F**) LC3, P62, and VEGF levels in BMECs from the GIT1 WT and KO mice after being treated with or without myelin debris for 72 h. N = 6 in each group. NS indicates no significance, ^*^p < 0.05, ^**^p < 0.01, ^***^p < 0.001.

Both *in vivo* and *in vitro* analyses revealed that GIT1 could effectively prompt MECs to engulf and degrade myelin debris. Numerous studies have confirmed that myelin debris is delivered and degraded through autophagy pathway in MECs [14, 24, 25]. Our previous research has demonstrated that GIT1 plays a key role in autophagy, which is tightly related to MECs uptake and the elimination of myelin debris [18–20, 26]. Thus, we hypothesized that GIT1 could prompt MECs to clear myelin debris via autophagy pathway. First, BMECs were transfected with the mRFP-GFP-LC3 adenovirus to detect autophagic flux. Yellow and red puncta were increased by autophagy and decreased by autophagy inhibition [27]. As shown in Figure 7A, 7B, few yellow and red puncta were observed and comparable between the GIT1 WT and KO groups without myelin debris treatment. However, having been stimulated with myelin debris for 72 h, both yellow and red puncta increased significantly in the GIT1 WT group, compared with those in the GIT1 KO group, suggesting that the knockout of GIT1 could reduce myelin debris-induced autophagic flux in BMECs. Western blot analysis was further used to confirm this result. We found that both the LC3-I conversion to LC3-II and the degradation of autophagy substrate P62, a hallmark of autophagy activation [28, 29], were inhibited in the GIT1 KO group, compared with the GIT1 WT group after being stimulated with myelin debris for 72 h (Figure 7C–7E). Collectively, these results suggested that GIT1 knockout could inhibit myelin debris-induced autophagy in BMECs *in vitro*. Moreover, in accordance with the above-mentioned results *in vivo* and *in vitro*, we also found that the weakened autophagy by GIT1 KO in myelin-laden BMECs could downregulate the expression of VEGF (Figure 7C–7F).

### Overexpression of GIT1 enhances myelin-induced autophagy in BMECs *in vitro*

**Figure 8:**
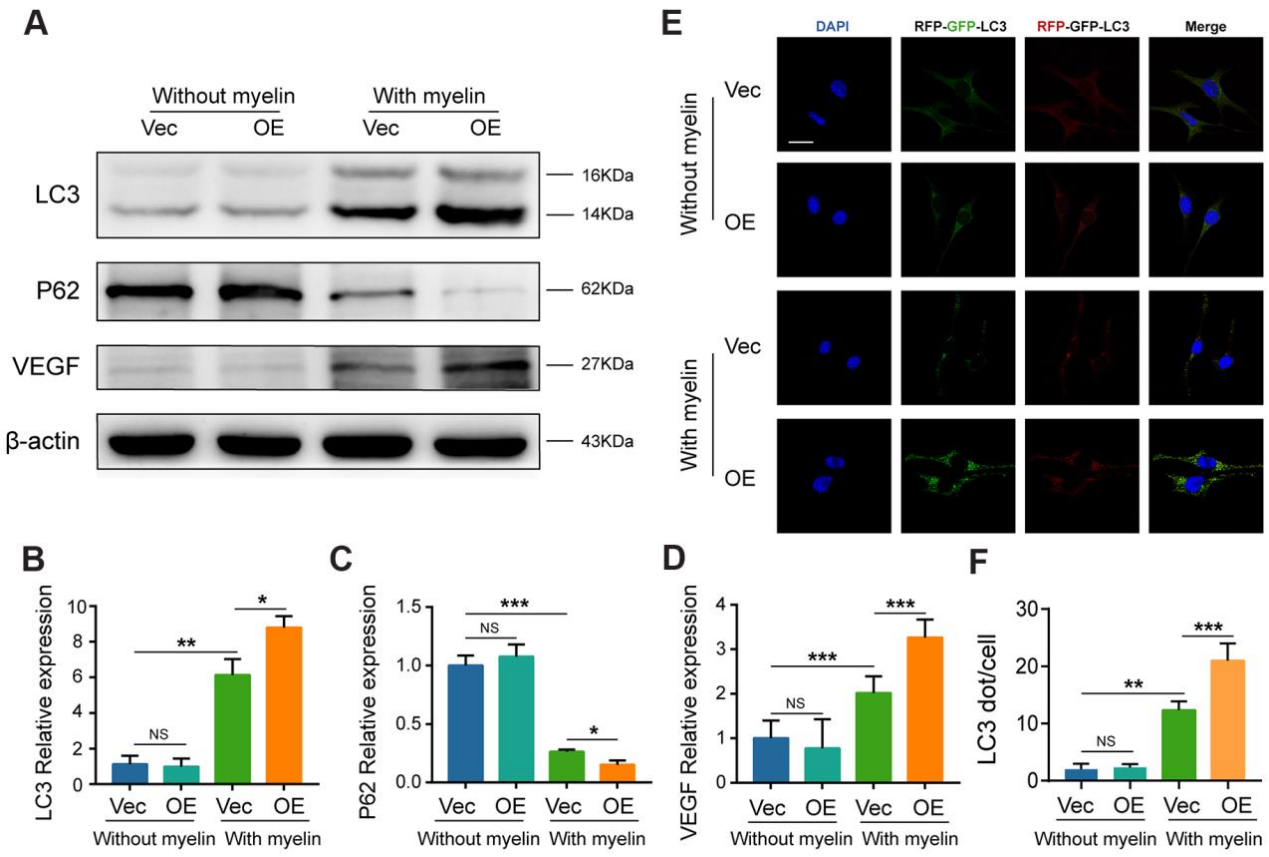
**Overexpression of GIT1 increases myelin-induced autophagy *in vitro*.** (**A**–**D**) LC3, P62, and VEGF levels in BMECs from the control and GIT1-OE groups after being treated with or without myelin debris for 72 h. (**E**) BMECs from the control and GIT1-OE groups were transfected with mRFP-GFP-LC3 adenovirus. The cells nuclei were stained using DAPI (blue). Bar, 10 μm. (**F**) Numbers of yellow and red puncta. N = 5 per group. NS represents no significance, ^*^p < 0.05, ^**^p < 0.01, ^***^p < 0.001.

As shown in Figure 5I, 5J and Figure 8A–8D, after being treated with myelin debris for 72 h, GIT1 overexpression significantly upregulated LC3-II level, accelerated substrate P62 degradation, increased VEGF expression and neutral lipid accumulation, compared to the control group (Figure 8A–8D and Figure 5I, 5J). What's more, having been stimulated with myelin debris for 72 h, the numbers of yellow and red dots were markedly increased in the GIT1-OE group, compared with those in the control group (Figure 8E, 8F). GIT1 overexpression could enhance myelin debris-induced autophagy, but this effect was not obvious in BMECs treated without myelin debris (Figure 8E, 8F).

## DISCUSSION

Myelin debris inhibits axonal regeneration and contributes to tissue damage after SCI [30] if not immediately ridded by BMDMs [8] and resident microglia [10, 31]. In this study, we found that MECs engulfed and degraded myelin debris within one week post-SCI, just as “amateur” phagocytes do. As an encouraging discovery, GIT1 prompts MECs to clear myelin debris through autophagy pathway, thus providing a promising therapeutic target for SCI.

Previous studies about myelin debris clearance mainly focused on macrophages and microglia. It needs at least one week for BMDMs to enter the injury site after SCI [8, 32], and microglia never show up in the lesion epicenter [10, 32]. In this study, we revealed that MECs gave rise to newly formed microvessels from one day, till their density came back to normal level at 7 days after SCI. Moreover, we also indicated that these new microvessels and MECs could swallow and degrade myelin debris at the injury site in the early stages. These observations moved us to speculate that MECs as “amateur” phagocytes could take over “professional” macrophages and microglia for myelin debris clearance shortly after SCI.

During autophagy, an isolated membrane envelopes the cytoplasmic cargo to form an autophagosome that transports the cargo to the lysosome for degradation [33, 34]. Autophagy has been defined as a mechanistic tool for MECs to clear myelin debris [14]. According to our previous studies, GIT1 participates in autophagy accomplished by osteoclasts under starvation condition [20], and neurons after ischemic-reperfusion injury [35]. These findings inspired us to ascertain the relationship between GIT1 and myelin debris clearance in MECs. We found that GIT1 promoted the clearance of myelin debris by BMECs. *In vitro*, we also showed that GIT1 deficiency inhibited myelin-induced autophagy in MECs. Collectively, our study indicated for the first time that GIT1 facilitated myelin debris clearance by MECs via autophagy pathway in SCI recovery.

MECs and its secreted angiogenic mediator (VEGF) could promote endothelial cells proliferation and angiogenesis after SCI [14, 36]. We previously demonstrated that GIT1 could activate PLC-γ and ERK1/2 in endothelial cells to maneuver VEGF expression via the GIT1- ERK1/2 axis [37]. Here, GIT1 was found to promote myelin debris clearance by MECs through autophagy, a process that may further stimulate MECs angiogenesis via increasing the expression of VEGF.

In our study, GIT1 KO mice were used, but we cannot rule out the possibility that GIT1 only exerts regulatory effect on MECs, and subsequent effects only appear after myelin debris has been cleared by MECs. Thus, further study should be carried out using conditional KO mice highly specific to MECs.

In conclusion, GIT1 could prompt MECs to clear myelin debris via autophagy and further stimulate MECs angiogenesis via upregulating VEGF. GITI may serve as a promising target for accelerating myelin debris clearance and improving SCI recovery.

## MATERIALS AND METHODS

### Cell culture and reagents

The primary mouse brain microvascular endothelial cells (BMECs) were isolated and cultured as previously described [38]. After isolation of mouse brains, the meninges-free forebrains were digested using 5 mg/mL collagenase CLS2 (Worthington Biochemical) in DMEM for 60 min, then centrifuged at 4° C for 20 min. The pellets were resuspended in 1 mg/mL collagenase/dispase (Worthington Biochemical) and incubated at 37° C for 60 min. After the final washing, the resultant cells were cultured in endothelial cell medium. The isolated BMECs purity was determined by staining with an anti-CD31 antibody (Abcam, Mouse, Monoclonal, ab24590) (Supplementary Figure 3A). Other antibodies used were anti-GIT1 (Novus, Mouse, Monoclonal, NBP2-22423), anti-β-actin (Abways, Mouse, Monoclonal, AB0011), anti-GFAP (Abcam, Mouse, Monoclonal, ab10062), anti-NF200 (Abcam, Rabbit, Monoclonal, ab207176), anti-MBP (Abcam, Rabbit, Polyclonal, ab40390), anti-VEGF (Abcam, Rabbit, Monoclonal, ab52917), anti-LC3 (CST, Mouse, Monoclonal, 83506), anti-P62 (CST, Rabbit, Monoclonal, 23214). The secondary antibodies were anti-rabbit Alexa Fluor 488 (Abcam, Goat, ab150077), anti-rabbit Alexa Fluor 594 (Abcam, Goat, ab150088), anti-mouse Alexa Fluor 488 (Abcam, Goat, ab150113), anti-mouse Alexa Fluor 594 (Abcam, Goat, ab150120). ELISA kits were VEGF (MultiSciences, 70-EK283/2) and MBP (Elabscience Biotechnology, E-EL-M0805c).

### Spinal cord injury

Homozygous GIT1 global knockout (KO) mice were generated on the C57/BL6 background as described previously [19, 35, 39]. Chimeric mice generated were backcrossed for more than seven generations. The GIT1 wild type (WT) littermates were taken as controls. With the approval of the Animal Committee of the First Affiliated Hospital of Nanjing Medical University, the animal experiments were carried out. We selected mice of 8–10 weeks to construct the SCI contusion models. After anesthetization, T10 was exposed. The contusion injury was created by a 5 g rod fallen from 6.25 mm upon the spinal cord, through the impactor (68097, RWD, USA). In the sham group, only laminectomy, but no contusion injury, was made.

### Basso Mouse Scale (BMS) behavioral analysis

After SCI, we used BMS to evaluate the motor function. Before the behavioral analysis, each mouse was trained in an open field [40]. Then, their behaviors were recorded by two independent researchers blinded to the trial at day 1, 3, 7, 14, 21, and 28 after SCI. On the BMS scale, 0 (no ankle movement) to 9 (complete functional recovery) was given according to the performance of hindlimb joint action, stepping coordination, paw location, toe clearance, and tail position.

### Electrophysiology

To assess the functional recovery after SCI, electromyography was employed to observe motor-evoked potentials (MEPs) of mice at day 28 after SCI according to previous research [41–43]. With a single square wave stimulus (0.5 mA, 0.5 ms, 1 Hz), the nerve conduction function of hindlimbs was examined by peak-to-peak amplitude.

### Magnetic resonance imaging (MRI)

After anesthetization, the mice were fixed. Using a small animal MRI system (Bruker BioSpec 7T/20 USR, Germany), the procedures were accomplished [43]. T2-weighted images were captured on the sagittal and axial planes by the ParaVision 6.0.1 (Bruker BioSpec, Germany).

### Tissue processing

At different time points after SCI, the mice were anesthetized, followed by transcardial perfusion with saline and then 4% paraformaldehyde (PFA). T6-L1 segments covering the lesion site were removed, post-fixed in PFA (4%, w/v) at 4° C for one day, dehydrated in a sucrose gradient (15% and 30% w/v), embedded in OCT, and longitudinally sectioned (thickness 10 μm).

### Histopathological analyses

### Spinal cord tissues morphology

In order to observe the gross morphology change of each spinal cord at day 28 post-SCI, the images were caught through a digital camera before the tissues section.

### H&E staining

At day 28 after SCI, the sections were stained using haematoxylin for 1 min, differentiated with 1% hydrochloric acid, and stained using eosin for 2 min. The identification of traumatic lesion area was determined by obvious tissue disarrangement and/or the loss of staining. The measurement of damage area in each section was performed by ImageJ software (National Institutes of Health). The lesion volume was acquired by the sum of total lesion area multiplied by distance between the sections.

### Oil Red O (ORO) staining

In order to present neutral lipid in spinal cord tissues and/or cultured cells, ORO staining was carried out. Frozen sections and/or fixed BMECs were dried in 100% propylene glycol (PG) and subsequently stained using 0.5% ORO solution at 60° C for 6 min. Having been soaked in 85% PG for 2 min and rinsed three times with distilled water, the slides were investigated with a microscope. The positive ORO area was obtained using ImageJ software.

### Immunofluorescence assessment

At the indicated time points, spinal cord tissues and/or cultured BMECs were fixed using PFA (4%, w/v) at room temperature, blocked in 10% bovine serum albumin (BSA), and incubated with primary antibodies at 4° C overnight. After three rounds of PBS washing, immunolabeled cells and/or tissues were incubated with the secondary antibodies for 50 min under darkness at room temperature. Then, the nuclei were stained using 4',6-Diamidino-2-Phenylindole (DAPI) (Thermo Fisher Scientific) and images were taken by a fluorescence microscope (AXIO Vert.A1 and Imager A2, Germany).

### Western blot analysis

Protein lysates were collected from BMECs and/or tissues covering the lesion site by lysis buffer, supplemented with protease and phosphatase inhibitor tablets (KeyGEN Biotechnology, China). Protein concentration was tested with Bradford method, separated by gel electrophoresis, transferred onto polyvinylidene difluoride (PVDF) membranes, blocked in 5% BSA, and incubated with corresponding antibodies overnight at 4° C. After incubation of membranes with the secondary antibody (Jackson ImmunoResearch, USA), bands were displayed using enhanced chemiluminescence (ECL) reagents, and their density was semi-quantified by ImageJ software.

### GIT1-overexpression plasmid construction and transfection

The plasmid containing GIT1-overexpression (OE) and a negative control plasmid were acquired from FulenGen Ltd., Co. (Guangzhou, China), then GIT1 was transfected into BMECs following the manufacturer’s instruction with empty vector cells as control. Western blot confirmed GIT1 overexpression efficacy.

### Myelin debris isolation and labeling

A rapid homogenization of the brains was performed using 0.32 M sucrose solution. Next, 0.83 M sucrose solution was added on the top. Having been centrifugated at 100,000 × g at 4° C for 45 min, the myelin debris at the interface of the two sucrose densities was collected [44]. Carboxyfluorescein succinimidyl ester (CFSE, Life Technologies, CA) was employed to label the myelin debris after 30 min incubation at room temperature.

### Myelin debris uptake assay

CFSE-labeled myelin debris was added to BMECs at different time points, till its concentration reached 1 mg/ml. To clean the remaining myelin debris, the cells were washed with PBS for 30 s. Myelin debris uptake was manifested with immunofluorescence, flow cytometry and ELISA.

### Flow cytometry

CFSE-labeled myelin debris was used to challenge BMECs for 72 h. Then, the remained debris was washed away. The cells were resuspended in PBS, then immediately detected with a flow cytometer (FACSVerse 8, BD).

### Enzyme-linked immunosorbent assay (ELISA)

To measure the engulfed myelin debris and how GIT1 regulates the expression of vascular endothelial growth factor (VEGF) in BMECs, the concentration of intracellular myelin basic protein (MBP) and the level of VEGF in the conditioned medium (CM) from the BMECs’s supernatant were measured using ELISA kits. Optical density was tested with a plate reader (ELx800, Bio-Tek).

### Tube formation assay

Having been thawed on ice, 50 μL of Matrigel was placed in 96-well plates and incubated for 30 minutes at 37° C. After Matrigel polymerized, BMECs (2 × 10^4^ cells/well) were added. After cell adherence was detected, conditioned medium (CM) from different groups (treated with or without myelin debris for 72 h) was added. As incubation terminated, a digital camera (NikonInc) was used to photograph the total tube length in each chamber.

### Wound-healing assay

BMECs were seeded into six-well plates (200,000 cells/well), till 80% confluence was achieved [45]. Next, a sterile pipette tip was introduced to scratch the confluent layers. After washing, CM from different groups (treated with or without myelin debris for 72 h) was added. At 0 h and 12 h after scratching, images were made.

### Cell viability assay

BMECs were seeded in a 96-well plate (2000 cells/well). After cell adherence was achieved, the supernatant was replaced with CM from different groups (treated with or without myelin debris for 72 h). Cell viabilities were tested at 0, 24, 48, and 72 h. Next, 10 μL of CCK8 was put into each well and incubated for 2 h at 37° C. Optical density values were acquired by a microplate reader at 450-nm wavelength.

### LC3 puncta quantification

BMECs were transfected with the mRFP–GFP–LC3 adenovirus (Hanbio Biotechnology Co., Ltd., Shanghai, China), then treated with or without myelin debris for 72 h. The nuclei were counterstained using DAPI. A Confocal Imaging System (Zeiss LSM710, Germany) was employed to take microphotographs. Yellow and red dots were quantified to evaluate autophagy formation and autophagic flux.

### Statistical analysis

Results are presented as mean ± standard deviation (SD) for at least four independent experiments. One-way or two-way analysis of variance was conducted if more than two groups were compared. Unpaired two-tailed student’s t-test was employed to make two-group comparisons. GraphPad software 7.0 and SPSS 19.0 were used for statistical analysis. Statistically significance was determined when p < 0.05.

## Supplementary Material

Supplementary Figures
