# Supplementary material for: GIT1 protects traumatically injured spinal cord by prompting microvascular endothelial cells to clear myelin debris
Source: Aging (Albany NY). 2021 Feb 17;13(5):7067–83. doi: 10.18632/aging.202560 (PMC7993661; doi:10.18632/aging.202560)
Supplement: Supplementary Figures [file aging-13-202560-s001.pdf]

SUPPLEMENTARY FIGURES

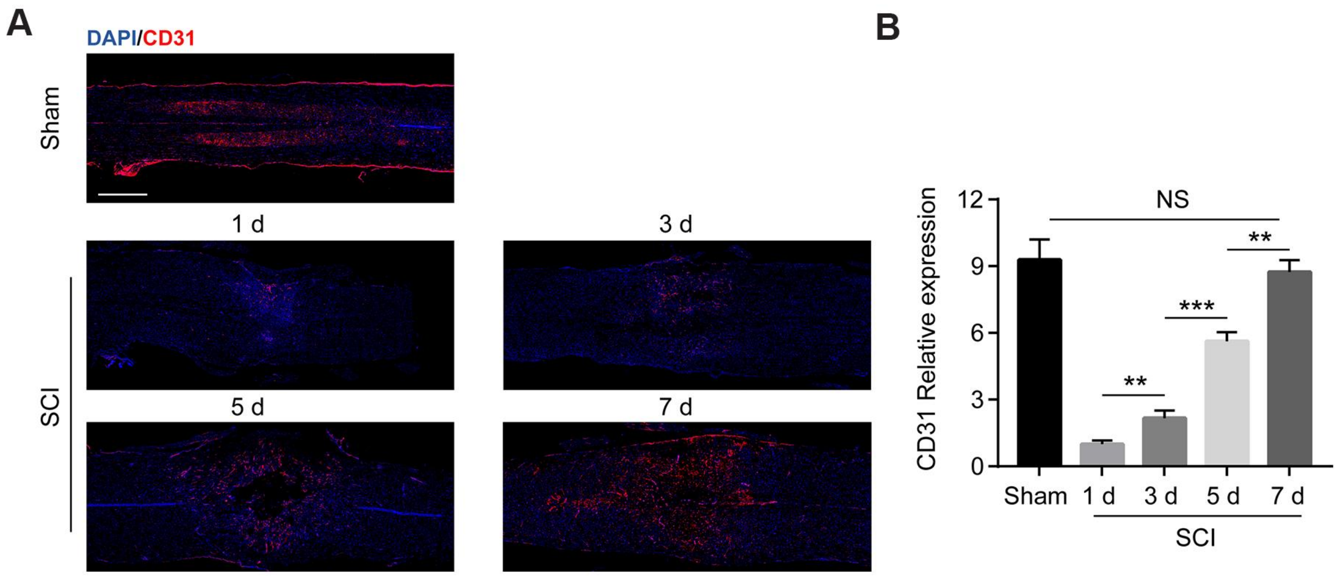

Supplementary Figure 1. Quantification of microvessels in spinal cords after SCI or sham surgery. (**A**, **B**)Representative immunofluorescence images for CD31 (red) and quantification at different time points after SCI or sham surgery. Nuclei were stained using DAPI (blue). Bar, 500 µm. N = 6 animals per group. NS represents no significance, $^{**}p < 0.01$, $^{***}p < 0.001$.

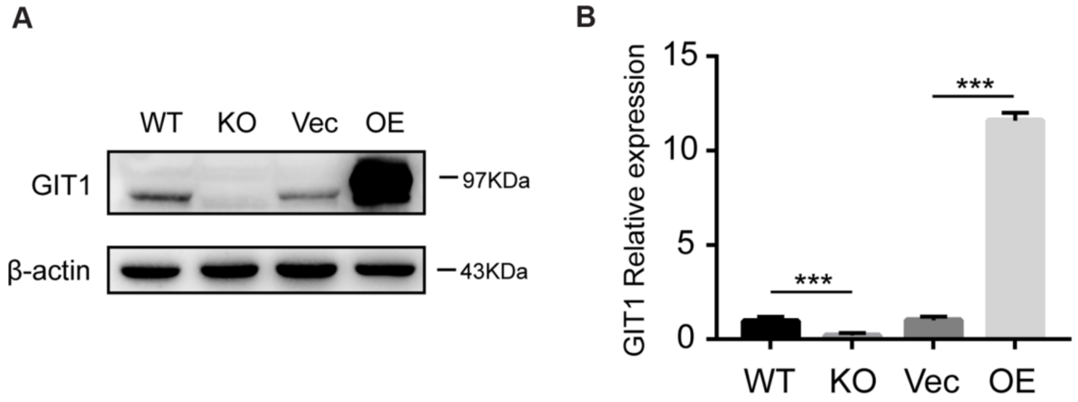

Supplementary Figure 2. The efficiencies of GIT1 knockout and overexpression. (**A**, **B**) The knockout and overexpression efficiencies of GIT1 in BMECs were confirmed by western blotting. N = 5 in each group. $^{***}p < 0.001$.

A

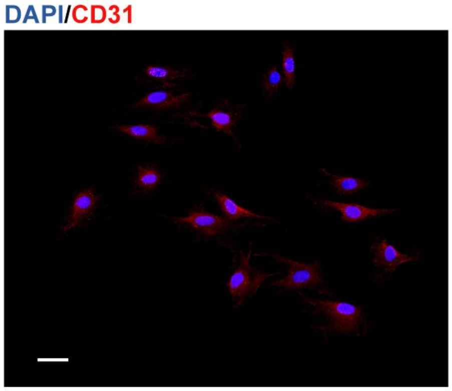

Supplementary Figure 3. The purity of isolated BMECs. (**A**) Anti-CD31 antibody staining identifying the purity of isolated BMECs. Nuclei were stained using DAPI (blue). Bar, 50 μm.
